# Improving Non-Line-of-Sight Identification in Cellular Positioning Systems Using a Deep Autoencoding and Generative Adversarial Network Model

**DOI:** 10.3390/s24196494

**Published:** 2024-10-09

**Authors:** Yanbiao Gao, Zhongliang Deng, Yuqi Huo, Wenyan Chen

**Affiliations:** 1 School of Electronic Engineering, Beijing University of Posts and Telecommunications, Beijing 100876, China; dengzhongliang@bupt.edu.cn (Z.D.); huoyuqi@bupt.edu.cn (Y.H.); 2 School of Mechanical Engineering and Automation, Northeastern University, Shenyang 110819, China; 2370098@stu.neu.edu.cn

**Keywords:** non-line-of-sight, generative adversarial network, autoencoder, localization

## Abstract

Positioning service is a critical technology that bridges the physical world with digital information, significantly enhancing efficiency and convenience in life and work. The evolution of 5G technology has proven that positioning services are integral components of current and future cellular networks. However, positioning accuracy is hindered by non-line-of-sight (NLoS) propagation, which severely affects the measurements of angles and delays. In this study, we introduced a deep autoencoding channel transform-generative adversarial network model that utilizes line-of-sight (LoS) samples as a singular category training set to fully extract the latent features of LoS, ultimately employing a discriminator as an NLoS identifier. We validated the proposed model in 5G indoor and indoor factory (dense clutter, low base station) scenarios by assessing its generalization capability across different scenarios. The results indicate that, compared to the state-of-the-art method, the proposed model markedly diminished the utilization of device resources and achieved a 2.15% higher area under the curve while reducing computing time by 12.6%. This approach holds promise for deployment in future positioning terminals to achieve superior localization precision, catering to commercial and industrial Internet of Things applications.

## 1. Introduction

Currently, location-based services are utilized in several aspects of our daily lives [1,2]. The demand for indoor positioning has exceeded the expectations of researchers. Accurate real-time location information breaks the barriers between virtual data and physical objects, which can revolutionize industries such as manufacturing, logistics, and navigation of large-scale public places, driving the Internet of Everything [3,4]. The development of artificial intelligence and robotics and the rise of new industries, such as autonomous healthcare and intelligent manufacturing, also rely on location-based services (LBSs) for technical support [5,6]. However, the reliability of LBS services depends on accurate positioning. Outdoor positioning technology relies mainly on established global navigation satellite systems (GNSSs) that have achieved submeter accuracy [7]. Nevertheless, due to object occlusion and multipath effects, the positioning accuracy of the GNSS is significantly degraded indoors and in urban canyons [8,9]. Thus, addressing blind spots in GNSS indoor positioning signal coverage is essential for seamless indoor–outdoor high-precision positioning and navigation [10,11].

Historically, wireless local area network-based positioning and navigation technologies such as Bluetooth [12], ultra-wideband [13], and Wi-Fi [14] garnered attention. However, scaling these systems from single buildings to cities incurs substantial network construction costs [15].

In contrast, 5G telecommunication infrastructure offers comprehensive signal coverage across most regions, including cities, suburbs, and rural areas [16]. Establishing 5G positioning systems presents cost advantages over other positioning methods, leveraging 5G’s inherent large bandwidth for seamless indoor–outdoor positioning [17].

Based on these capabilities, the 3rd Generation Partnership Project (3GPP) has incorporated support for radio interface technology-dependent positioning in the Rel-16 version of the 5G protocol [18]. In addition, with the recent development of the large-scale antenna system, also known as massive multiple-input multiple-output (massive MIMO), 5G positioning systems can be implemented in various scenarios, such as indoor spaces and factories [19]. Massive MIMO provides additional degrees of freedom by utilizing the spatial and angular domains of the propagation channel in combination with temporal measurements. This enables a 5G-based positioning system to support multiple localization techniques, including downlink time difference of arrival, downlink angle of departure, uplink time difference of arrival, uplink angle of arrival (AoA), and multicell round-trip time, which ensure higher positioning accuracy than that obtained with a long-term evolution (LTE)-based positioning system [20].

**Figure 1:**
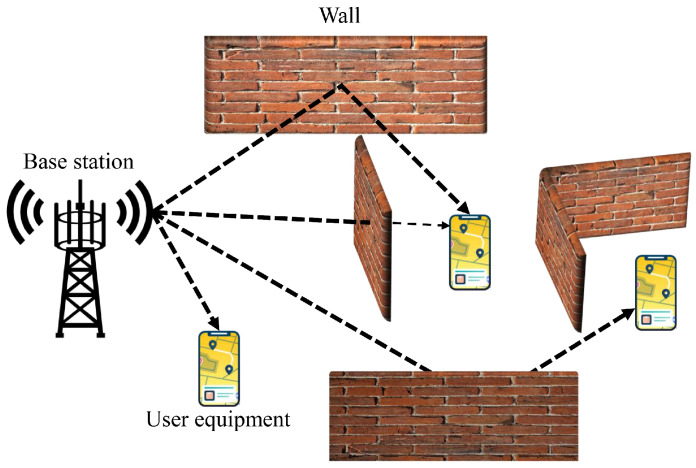
Possible modes of signal propagation.

Notably, the trilateration- and triangulation-based localization methods mentioned above achieve optimal performance when the signal propagates in line-of-sight (LoS) environments, i.e., no obstacles are blocking signal propagation [21]. However, in an actual localization scenario, obstacles may exist and their positions may change. When the signal encounters these obstacles in the propagation process, diffraction, reflection, and scattering are likely to occur, resulting in different deviations in the localization and angulation received by the positioning terminal, which seriously affects the positioning accuracy [22]. Fluctuations in observations challenge real-time localization reliability, potentially leading to security vulnerabilities [23]. Figure 1 illustrates various modes of signal transmission.

Notably, according to the functional specifications of user equipment (UE) positioning in a next-generation radio access network (NG-RAN), known as TS 38.305 [20], most instances of location solving occur in the location management function (LMF) [24]. Even cell phones with sufficient computational power can assist in location settlement. During this process, the LMF receives measurements uploaded by the UE or gNodeB (gNB), which can be used to extract features for non-line-of-sight (NLoS) identification.

Therefore, many scholars have examined various NLoS identification and suppression methods, such as the residual-weighted geometric hyperbolic (RWGH) method [25], Neyman–Pearson detection method [26], and Bayesian sequence statistics method [27], each of which has its merits and limitations. We will further discuss these issues in the related works section.

The most obvious limitation is that the probability distribution models struggle to represent the NLoS distribution accurately, particularly considering its variability with indoor objects. In contrast, LoS propagation uniformly follows a Rician distribution [28]. Moreover, as communication standards progress, multimodal and multi-task learning have become a crucial pathway for the enhancement of machine learning in communication technology. Consequently, machine learning functionalities will undoubtedly become a common component of the forthcoming generation of communication systems. Furthermore, non-line-of-sight identification in cellular positioning systems is intricately linked to multitasking and multimodality. Multitasking- and multimodality-based machine learning techniques hold significant reference value for the intelligent prediction of instances [29,30,31]. Therefore, we proposed an NLoS identification method based on training LoS samples. This approach allows the network to accurately recognize the LoS by extracting the latent features of the LoS samples and identifying the NLoS as outliers for precise NLoS identification. This scheme improves network generalization and reduces inference time and storage requirements, facilitating the adoption of positioning terminals.

## 2. Related Works

In this section, we categorize the existing NLoS recognition methods into two types: traditional NLoS recognition methods and machine learning (ML)-based NLoS recognition methods. We briefly explain the principles of these methods and highlight their shortcomings. Eventually, we use this analysis to develop the proposed method.

### 2.1. Conventional Methods

Traditional methods for NLoS identification primarily involve two approaches: the first is gathering data from several signal sources simultaneously and assessing whether a signal source exhibits NLoS propagation by cross-analysis of a designated selection of signal sources for comparison. The alternative method is collecting numerous measurements from a singular signal source, constructing a probability distribution, and employing the subordinate relationship between the observed data and the distribution to ascertain whether it signifies NLoS propagation.

The RWGH method is the standard approach for aggregating data from various signal sources to assess if a limited number of signal sources are in the NLoS propagation stage. The RWGH approach utilizes the estimated location coordinates to calculate observation residuals and evaluates these residual values to determine whether an observation is under the NLoS propagation condition [25]. The advantage of this method is that prior knowledge is not required. Ref. [32] leveraged the Cayley–Menger determinants within the framework of the distance geometry theory to ascertain NLoS propagation across three channels within each group, subsequently identifying specific LoS and NLoS anchors through an exhaustive traversal of the detection outcomes across all groups.

Nonetheless, the technique for aggregating various signal sources imposes stringent demands on the user’s surroundings and is capable only of identifying anomalous NLoS propagation, which is inherently unstable. Consequently, it is more prevalent to formulate a probability distribution for the propagation of a singular signal source and utilize this distribution to ascertain the NLoS propagation of the signal source [33]. Aghaie utilized the generalized likelihood ratio test method, postulating that the measurement and NLoS errors follow distinct probability distributions [26]. The probability that different types of channel information belong to different distributions was calculated according to the Neyman–Pearson criterion. NLoS identification was conducted by adjusting the false alarm and detection probabilities. However, the efficacy of this method is heavily dependent on the parameter settings, indicating limited generalization capability. Wang et al. adopted the Bayesian sequential test method, wherein the probabilities are updated after each new data collection and the presence of NLoS is determined based on previous knowledge and current evidence [27]. Similarly, Yan et al. used Bayesian sequential testing to ascertain whether the current propagation is NLoS and applied a modified Kalman filter to smooth the NLoS measurement data, thereby reducing the NLoS forward error [34].

The aforementioned conventional techniques for determining probability distributions frequently encounter the same issue: the information dimension of the probability predominantly comprises the time of arrival and the angle of arrival. This information significantly relies on the estimation methods for distance and angle, rendering it infeasible to utilize propagation data of higher dimensions to assess the probability distribution. Estimating a probability distribution simultaneously requires a predetermined type of probability distribution, and identifying the exact probability distribution associated with NLoS is challenging. Recognizing a probability distribution will profoundly influence the estimation result. Consequently, researchers have contemplated employing machine learning techniques for NLoS identification.

### 2.2. Machine Learning Methods

Given that signal propagation can fundamentally be categorized into LoS and NLoS, the identification of NLoS can inherently be framed as a binary classification problem [35,36,37]. Consequently, the introduction of ML techniques to address the issue of NLoS identification is feasible [38]. The primary consideration is the selection of the network inputs [39]. Numerous studies have previously utilized the estimated channel characteristics as the inputs, including maximum correlation magnitude, energy decay, peak decay exponent, root-mean-square delay spread, mean excess delay, Rician K-factor, kurtosis, skewness, and total received power [40,41,42,43,44,45,46,47,48]. With the parameters mentioned above as the network inputs, researchers initially considered employing the random forest methodology to identify NLoS propagation [49,50]. This method constructs multiple decision trees for network training by randomly selecting samples and training parameters. In the testing phase, decisions are made by feeding inputs to multiple decision trees, and multiple decisions are weighted to determine whether the signal propagation is LoS or NLoS [51,52]. Similarly, methods based on Support Vector Machines (SVMs) for NLoS identification have been considered [53,54,55]. Barral et al. utilized the received signal strength and range as features for NLoS discrimination using an SVM [56].

The method mentioned above, although effective, essentially addresses only the issue of multi-parameter threshold selection for judgment, whereas the overall recognition performance of the NLoS continues to be constrained by the inherent limitations of these features. Consequently, the academic community has considered directly inputting the raw channel state information, such as the channel impulse response (CIR), into the networks to allow them to autonomously learn the underlying features to realize NLoS identification [57,58,59]. Zhu et al. [60] defined a lightweight convolutional neural network (CNN) architecture for NLoS identification. Si et al. [61] combined multilayer perceptrons (MLPs) with CNNs to achieve enhanced performance. Kong developed the CNN framework and utilized the dung beetle optimizer (DBO) to design an improved DBO-CNN that employed circle chaotic mapping, non-uniform Gaussian mutation strategies, and multi-stage perturbation strategies to optimize exploration capabilities, thereby improving the F1-Score by 3.31% [62]. However, the primary issue with this method is the requirement for a substantial number of labeled samples. Equal numbers of NLoS and LoS channel samples are required to learn the features of the NLoS and LoS channels adequately. Collecting NLoS propagation samples that cover nearly all scenarios during measurements is impractical, and their application across different scenes presents even more significant challenges.

Based on the issues outlined above, one possible solution is a one-class neural network built by training solely with LoS dataset samples, which enables deep neural networks to exhaustively learn the latent features of a single category of samples and construct hyperplanes to differentiate between LoS and NLoS [63,64]. However, this method is computationally complex and updates are slow. Another approach utilizes an autoencoder (AE) to learn the latent features of the LoS samples for efficient encoding [65,66]. The training method involves training a pair of encoders and decoders simultaneously; the encoder first encodes the samples and then decodes them using the decoder [67]. The reconstructed samples are compared with the original samples to construct a loss function for model training. This method can fully extract the features of the LoS samples [68]. Moreover, for NLoS data that do not belong to LoS, the samples reconstructed through encoding and decoding will generate a significant reconstruction error, thereby revealing themselves as NLoS samples [65,69,70,71]. However, the selection of an appropriate discrimination threshold has always been challenging [72]. To this end, some researchers have proposed using the kernel density estimation (KDE) method to estimate the probability density distribution of LoS samples.

By calculating the probability that a test sample belongs to a set of LoS samples, we can determine whether the sample is an LoS sample. This method can reduce the difficulty in threshold selection to a certain extent and is highly accurate. However, a major issue with this method is the need to cache many LoS samples in memory to complete the KDE, thus significantly limiting its practicality.

### 2.3. Contributions

In response to the issues outlined above, we propose a novel method for NLoS identification in the context of 5G positioning systems. The method aims to address the decline in positioning performance, caused by signal propagation under NLoS conditions. The main contributions of this study are as follows:

- We improved the basic multipath channel propagation model for a uniform planar array (UPA)-based 5G transmission system. The traditional direction-of-arrival (DoA)-based positioning system model solely focuses on the phase shift of the wavefront reaching each array element without considering the signal content. Therefore, this model is limited to single-frequency or signals centered around a single frequency, failing to meet the analysis requirements of Orthogonal Frequency Division Multiplexing (OFDM) systems. In this study, we analyzed the delay angle of the signal arriving at each array element and derived the phase shift of the position of the array element across all frequency points within the transmission bandwidth. Specifically, in OFDM systems, the improved model can analyze the impact of the delay angle of the signal arriving at each array element on every subcarrier. Finally, we constructed a channel frequency response (CFR) matrix of the UPA-based 5G transmission system.
- We refer to the processing method for the CFR in the fingerprint localization system and transform the CFR into an angle-delay channel power matrix (ADCPM) as the input to the network so that the network can learn the potential features of the LoS distribution more intuitively [73]. However, owing to variation in the UE location, the ADCPM obtained by the transformation is sparse. Moreover, the peak positions always differ, which significantly affects the learning of other potential features by the network. To this end, we propose a deep autoencoder with channel transformer (DACT) architecture, which utilizes an AE for feature extraction of the ADCPM, introduces the spatial transformer network (STN) to transform the ADCPM, and constrains the loss function related to the difference in the encoder output before and after the transformation, making the network less sensitive to the peak positions of the ADCPM and more sensitive to the extraction of other potential features of LoS propagation.
- We designed a generative adversarial network (GAN) oriented toward NLoS identification by feeding the AE-encoded ADCPM into the discriminator of the GAN to determine whether it belongs to NLoS propagation. Because the output of the discriminator is a probability, a threshold of 0.5 can be set directly, which solves the problem of threshold selection in traditional NLoS recognition methods. Second, this method can generate various samples from random noise during the generator training process, which allows the discriminator to explore more possibilities without the participation of NLoS samples. This further improves the accuracy of LoS feature extraction and robustness of NLoS identification when compared with the results of pure learning of LoS samples using only the AE. Unlike the KDE method, the proposed method does not need to load training samples in the testing phase, which significantly reduces memory consumption. Finally, because the inputs need to pass through only the encoder and discriminator in the testing phase, the prediction speed is significantly improved when compared with that of the KDE method, which provides strong support for deploying the NLoS recognition method in the localization terminal.
- We simulated the signaling scenarios for 5G sounding reference signals (SRSs) in indoor offices and factories (dense clutter low base station) based on the 3GPP TS 38.901 standard and verified the effectiveness of the proposed method across the scenarios by training in one scenario and validating in the other scenarios.

The remainder of this paper is organized as follows: Section 3 presents the system modeling and introduces the CFR for a 5G positioning system, elucidates the data preprocessing, namely, transforming the CFR into ADCPM, and describes the details of the designed deep autoencoding channel-transformed generative adversarial network (DACT-GAN) network architecture and construction of the related loss functions. Section 4 introduces the setup for environmental simulation including the testing platform, specific parameters of the network model, and state-of-the-art (SOTA) methods. Section 5 compares the proposed method with the SOTA methods, and quantifies the superiority of the proposed method. Finally, we conclude the paper in Section 6.

## 3. Methodology

In this section, we presented the system modeling, and addressed the NLoS identification issue in terms of data acquisition and labeling, culminating in the adoption of a GAN discriminator as the NLoS identifier. Subsequently, to facilitate a more profound feature extraction, we incorporated the angle-delay domain channel power matrix as the input to the NLoS identification network and delineated the transformation process from the CFR to the ADCPM. Accordingly, we defined the NLoS identification network proposed in this study as a DACT-GAN. Finally, the network loss function was introduced.

### 3.1. System Model

We consider 5G-based wireless positioning systems, where 5G signals propagate as electromagnetic waves over air. We can define a 5G-based wireless positioning system as a linear time-invariant system. Consider an uplink where a single-antenna UE broadcasts 5G SRSs to a single-antenna 5G base station (BS); the propagation channel can be modeled as:

(1) $$h\left(t\right)=\sum_{p=0}^{P-1}\alpha_{p}\delta \left(t-\tau_{p}\right).$$

Due to phenomena such as reflection, refraction, and diffraction that may occur as the signal propagates from the UE to BS, multiple propagation paths may be generated, leading to a multipath effect. In this study, we define the number of multipath effects produced during the signal propagation process as *P*. Specifically, we define the attenuation caused by space on the signal in each propagation path as α and the delay as τ. Typically, without blocking the first-arrival path, we consider $\tau_{0}$ to be the direct path.

**Figure 2:**
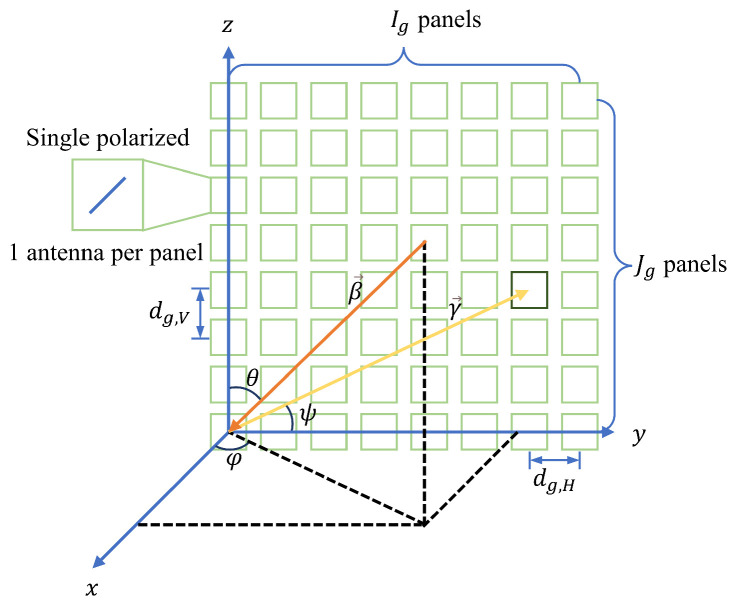
UPA with antenna panels, each consisting of one single polarized antenna element. The arrived signal’s AoA is decomposed into azimuth and elevation angles.

In contemporary deployments, massive MIMO constitutes a pivotal component of 5G technology [74,75]. Within the scope of this study, we postulate that the BS is equipped with a uniform rectangular panel array encompassing $J_{g}\times I_{g}$ panels, where $I_{g}$ denotes the number of panels per row and $J_{g}$ denotes the number of panels per column. Each antenna panel is uniformly spaced horizontally and vertically; $d_{g,H}$ and $d_{g,V}$ denote the horizontal and vertical panel spacing, respectively. To focus on the identification of the NLoS conditions, we assume that each panel comprises a single element and that these antenna elements are single-polarized. Consequently, the total number of antenna elements in the UPA can be expressed as $J\times I=J_{g}\times I_{g}$. For visual reference, Figure 2 illustrates the UPA.

The arrival times of the signals at various antennas differ because of the different locations of the multiple antennas of the UPA. As shown in Appendix A, the far-field assumption is valid within the context of this study. Consequently, for UPAs, the propagation of 5G signals can be equivalently modeled as plane waves, indicating a uniform DoA across all array elements. Taking Figure 2 as an example, using the panel at the origin as the baseline panel, one can compute the DoA-related time delay, $\tau_{\mathrm{DoA}}$, for each panel within the UPA relative to the baseline panel. The method for calculating $\tau_{\mathrm{DoA}}$ of any panel in the UPA relative to the baseline panel is accomplished by constructing a vector $\vec{\gamma }$ directed from the baseline panel toward that panel; the magnitude of this vector and angle ψ between this vector and DoA vector are determined. These parameters facilitate the computation of the propagation delay distance, $d_{\mathrm{DoA}}$, of the electromagnetic wave, converted into a temporal unit for deriving the $\tau_{\mathrm{DoA}}$.

Based on the established parameters, the vector representing the position of the panel at the *j*-th row and *i*-th column relative to a standard reference panel is articulated in Cartesian coordinates as $\vec{\gamma_{i,j}}=\left(0,id_{g,H},jd_{g,V}\right)$. Furthermore, in the context of signal propagation, the direction of arrival is typically represented by the azimuth angle ϕ and elevation angle θ. Aiming to model the direction of arrival as a unit vector $\vec{\beta }$, we employ the conversion relationship between the polar and Cartesian coordinate systems to ascertain the Cartesian coordinates of $\vec{\beta }$:

(2) $$\begin{array}{cc} x & =\sin\theta \cos\phi \\ y & =\sin\theta \sin\phi \\ z & =\cos\theta \end{array}$$

The delay associated with wave arrival can be equated to the projection of $\vec{\gamma_{i,j}}$ in the direction of arrival. Given that the direction of arrival has been modeled as a unit vector $\vec{\beta }$, calculating the delay $d_{\mathrm{DoA},i,j}$ is effectively equivalent to determining the dot product between $\vec{\gamma_{i,j}}$ and $\vec{\beta }$, as follows:

(3) $$d_{\mathrm{DoA},i,j}=\vec{\gamma_{i,j}}\cdot \vec{\beta }=id_{g,H}\sin\theta \sin\phi +jd_{g,V}\cos\theta .$$

Then, the delay $\tau_{\mathrm{DoA},i,j}=d_{\mathrm{DoA},i,j}/c$. In summary, for each array element of the UPA, we can derive its CIR as:

(4) $$h_{i,j}\left(t\right)=\sum_{p=0}^{P-1}\alpha_{p}\delta \left(t-\tau_{p}-\tau_{\mathrm{DoA},i,j}\right).$$

Furthermore, because 5G signals are digital, we consider discrete systems for reception. Let the sampling interval be $T_{s}$. Then, the above equation can be expressed as:

(5) $$h_{i,j}\left(nT_{s}\right)=\sum_{p=0}^{P-1}\alpha_{p}\delta \left(nT_{s}-\tau_{p}-\tau_{\mathrm{DoA},i,j}\right).$$

If expressed as a sequence, the above equation can be further reduced to:

(6) $$h_{i,j}\left(n\right)=\sum_{p=0}^{P}\alpha_{p}\delta \left(n-m_{p}-m_{\mathrm{DoA},i,j}\right).$$

where $m_{p}=\left\lfloor \frac{\tau_{p}}{T_{s}}\right\rfloor$ is a quantized representation of the multipath delay and $m_{\mathrm{DoA},i,j}=\left\lfloor \frac{\tau_{\mathrm{DoA},i,j}}{T_{s}}\right\rfloor$ is a quantized representation of the relative delay of the antenna panel.

When considering the CIR matrix h characterized by the dimensions I×J×N, a simplification is proposed for ease of understanding. This entails consolidating the matrix along the *J* dimension, effectively reducing the two-dimensional matrix formed by the *I* and *J* dimensions to a one-dimensional matrix $h_{v}$. The correspondence between the elements is as follows:

(7) $$h_{v}\left(i\times J+j,n\right)=h\left(i,j,n\right),$$

where i=0,1,2,…,I−1, j=0,1,2,…,J−1, n=0,1,2,…,N−1. Thus, the dimensions of the reduced matrix $h_{v}$ are IJ×N.

The presence of multipath effects leads to frequency-selective fading during signal propagation. The essence of 5G systems lies in their foundation as orthogonal frequency-division multiplexing (OFDM) systems, where the signal design is predominantly executed in the frequency domain. Given these considerations, transforming the CIR $h_{v}$ into the CFR $H_{v}$ is imperative. This transformation requires the application of a discrete Fourier transform (DFT) to $h_{v}$:

(8) $$\begin{array}{cc} H_{v}\left(i\times J+j,k\right) & =\sum_{n=0}^{N-1}h_{v}\left(i\times J+j,n\right)e^{-j2\pi \frac{k}{N}n}\\ \; & =\sum_{n=0}^{N-1}\sum_{p=0}^{P-1}\alpha_{p}\delta \left(n-m_{p}-m_{\mathrm{DoA},i,j}\right)e^{-j2\pi \frac{k}{N}n}\\ \; & =\sum_{p=0}^{P-1}\alpha_{p}e^{-j2\pi \frac{k}{N}\left(m_{p}+m_{\mathrm{DoA},i,j}\right)} \end{array}$$

In the 5G localization system, $N=N_{f}$, where $N_{f}$ is the order of the Fourier transform of the unit OFDM symbols. Its value is determined by the number of subcarriers $N_{c}$, which is related to $N_{f}$ as $N_{f}=2^{\left\lceil \log_{2}\left(N_{c}\right)\right\rceil }$. Furthermore, because the number of subcarriers per resource block (RB) is fixed at 12, the number of subcarriers $N_{c}$ again depends on the number of RBs allocated to the UE by the BS. In summary, *N* depends on the number of RBs.

### 3.2. Problem Formulation

In practical measurements, collecting samples from various NLoS scenarios is infeasible. Consequently, the accuracy of supervised learning using the collected NLoS/LoS samples is debatable. Even if a model achieves high scores within the test set of a study, its precision and applicability may be challenged upon its introduction to new environments.

Therefore, one solution is to extract only the features of the LoS to discriminate the NLoS from mismatched features. Thus, we placed only the LoS data in the training set, allowing the model to fully extract the potential features of the LoS samples. However, to verify the effectiveness of the extracted features in the validation set, we collected a certain number of NLoS samples from the same scene.

In addition, to validate the cross-scene adaptability of the proposed method, we selected the data measured under different scenarios. In summary, the primary goal of the model is to fully extract the features of the LoS, followed by learning the method and basis of discrimination based on these features.

### 3.3. Network Inputs

In Section 3.1, we derived the CFR matrix, denoted as $H_{v}$, with dimensions of $IJ\times N_{f}$. Concurrently, it was established that $N_{f}\geq N_{c}$, where the discrepancy between the two values was attributed to the insertion of zeros during the Fourier transform process. Moreover, within the OFDM modulation procedure, the system translocated the zero-frequency component from the center to the start of the spectrum. Consequently, reconstructing the original information necessitated an initial cyclic shift in $H_{v}$ followed by the removal of the inserted zeros. The resultant matrix $H_{c}$ is defined as:

(9) $$H_{c}\left(i\times J+j,k\right)=\left\{\begin{array}{c} H_{v}\left(i\times J+j,N_{f}-\frac{N_{c}}{2}+k\right),\\ k=0,1,2,\cdots ,\frac{N_{c}}{2}-1\\ H_{v}\left(i\times J+j,k-\frac{N_{c}}{2}\right),\\ k=\frac{N_{c}}{2},\frac{N_{c}}{2}+1,\frac{N_{c}}{2}+2,\cdots ,N_{c}-1 \end{array}\right.$$

When designing OFDM systems, researchers have considered that multipaths can lead to intersymbol interference (ISI) in the propagation of signals, which can severely damage the orthogonality between the subcarriers. Therefore, OFDM systems add protection intervals before successive symbols to limit the ISI, typically by adding a cyclic prefix (CP). During the development of the 5G protocol, the 3GPP specified two types of CPs: normal and extended. In this study, we used a normal CP, defined as:

(10) $$N_{\mathrm{CP},l}^{\mu }=\left\{\begin{array}{cc} 144\kappa \cdot 2^{-\mu }+16\kappa & l=0\;\mathrm{or}\;l=7\cdot 2^{\mu }\\ 144\kappa \cdot 2^{-\mu } & l\neq 0\;\mathrm{and}\;l\neq 7\cdot 2^{\mu } \end{array}\right.$$

where *l* denotes the OFDM symbol index relative to a reference; the parameter μ represents the subcarrier spacing configuration, with the corresponding subcarrier spacing being $\Delta f=2^{\mu }\cdot 15\;\mathrm{kHz}$. The variable κ is a constant, signifying the ratio between the basic time units for LTE and 5G, valued at 64. For the convenience of subsequent derivations, we assume that the length of the CP exceeds the maximum delay produced by multipath propagation. Furthermore, we define a constant $N_{t}=144\kappa \cdot 2^{-\mu }$ as the upper limit for the delay dimension in the following ADCPM.

Based on the available knowledge, the most intuitive manifestation of multipath is the diversity of the arrival time delay and angles. To further optimize the feature extraction for the NLoS, we transform the $H_{c}$ matrix into the ADCPM matrix P. To this end, we define the phase-shifted DFT matrices $V_{I}={\left[v_{I}\left(r,s\right)\right]}_{I\times I}$ and $V_{J}={\left[v_{J}\left(r,s\right)\right]}_{J\times J}$, where:

(11) $$v_{I}\left(r,s\right)=\frac{1}{\sqrt{I}}e^{-j2\pi \frac{r\left(s-\frac{I}{2}\right)}{I}}\;,$$

(12) $$v_{J}\left(r,s\right)=\frac{1}{\sqrt{J}}e^{-j2\pi \frac{r\left(s-\frac{J}{2}\right)}{J}}.$$

Then, we consider the submatrix of dimensions $N_{c}\times N_{\mathrm{CP}}$, obtained from the inverse DFT matrix $F^{-1}$, where $f^{-1}\left(r,s\right)=\frac{1}{N_{c}}e^{j2\pi \frac{rs}{N_{c}}}$. In summary, we can initially derive the angle-delay channel response matrix (ADCRM) as:

(13) $$G=\frac{1}{\sqrt{IJN_{c}}}\left(V_{I}^{H}⊗V_{J}^{H}\right)H_{c}F^{-1},$$

where $V_{I}^{H}$ and $V_{J}^{H}$ denote the conjugate transpose of $V_{I}$ and $V_{J}$, respectively, and ⊗ represents the Kronecker product between the two matrices. However, its complex form renders the matrix less intuitive for neural networks. Thus, it is necessary to further transform the ADCRM into an ADCPM as:

(14) $$X=E\left\{G⊙G^{*}\right\},$$

where E and ⊙ represent the expectation of the quantity of interest and Hadamard product between the two matrices, respectively.

Owing to its intuitive reflection of the arrival angles and delays, the ADCPM can more directly reveal the characteristics of the NLoS. Simultaneously, we demonstrate that the ADCPM is sparse and the elements of the matrix are real numbers, making it more suitable for feature extraction using CNN-type networks than CFR. Therefore, this study employed the ADCPM as the input to the NLoS identification network.

### 3.4. DACT-GAN

**Figure 3:**
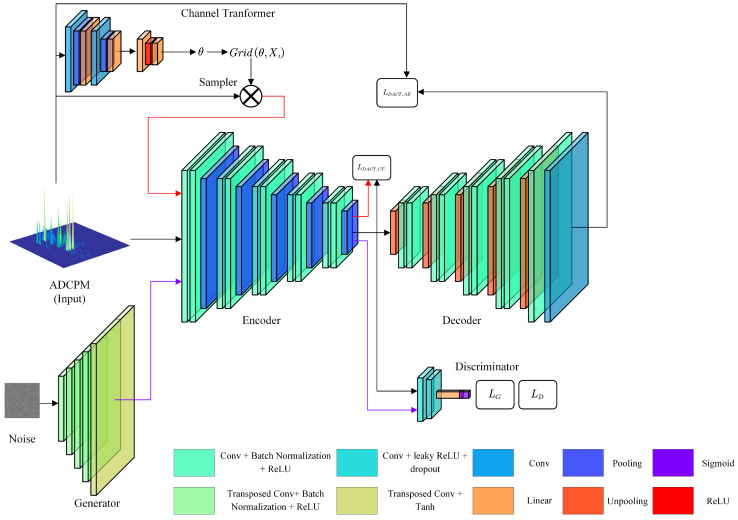
Structure of the proposed Deep Autoencoding Channel-Transformed Generative Adversarial Network (DACT-GAN) and components of the loss function to be computed.

The generalized NLoS identification method based on DACT-GAN proposed in this study consists of three main components: an AE, a channel transformer ($C_{T}$), and a GAN. A schematic of the model is shown in Figure 3. In the center of the figure is AE, which includes an encoder ($E_{n}$) and a decoder ($D_{e}$). $E_{n}$ is responsible for extracting the latent core features of ADCPM. The $E_{n}$ attaches the $C_{T}$, which performs a spatial transformation on the input samples and then encodes them. The loss function combines the two encoding results to reduce the influence of peak position on feature extraction. The $D_{e}$ is used to pair with the $E_{n}$, construct the loss function, and complete unsupervised learning. After ADCPM encoding is complete, the encoded samples are input into the GAN discriminator (*D*) to determine whether they are LoS samples or NLoS samples. The GAN generator (*G*) is responsible for generating fake ADCPM samples, passing them through the encoder, and comparing them with the real encoded samples to complete the joint training of *G* and *D*. Note that during testing, NLoS identification only requires the $E_{n}$ and *D* to complete the task. Hence, the actual operating rate is very high.

For convenience, we denote the ADCPM samples used for training as X, where the i-th sample is $X_{i}$.

The AE facilitates model training by minimizing the reconstruction error [76]. Specifically, the $E_{n}$ has layers with progressively decreasing dimensions and is tasked with extracting the latent core features of the sample $X_{i}$, making it exceptionally suitable for NLoS identification. We use Equation $z_{i}=E_{n}\left(X_{i}\right)$ to denote the encoding procedure that the encoder performs on the ADCPM samples, where $z_{i}$ is the encoded form of $X_{i}$.

The decoder is symmetrical to the encoder, which is responsible for restoring the encoded sample $z_{i}$ to its original form. If the core feature is not satisfied, the sample $\hat{X_{i}}$ recovered by the decoder will be far from the original sample $X_{i}$. In this study, we utilized this feature to extract the latent features of the ADCPM and thoroughly learn the features of the LoS to accomplish NLoS identification. We use Equation $\hat{X_{i}}=D_{e}\left(z_{i}\right)$ to denote the process by which the decoder reconstructs $z_{i}$ into $\hat{X_{i}}$.

However, the construction of an NLoS identification database requires measurement data from multiple positions within a scene to various BSs, resulting in inevitable variations in the distance and arrival angles between the BS and UE. Additionally, according to the definition of the ADCPM, under LoS conditions, the ADCPM accentuates the peak of the first-arrival path. Consequently, the features extracted by the AE inherently consider the position of the peak in the ADCPM, which is counterproductive for NLoS identification. To address this, we designed a $C_{T}$ that modifies the original sample $X_{i}$ before feature extraction by the encoder, resulting in $X_{i,c}$. This $X_{i,c}$ is then encoded as $z_{i,c}$. This approach aimed to minimize the discrepancy between $z_{i,c}$ and $z_{i}$, thereby reducing the effect of the peak position on the AE encoding. We use Equation $X_{i,c}=C_{T}\left(X_{i}\right)$ to denote the spatial transformation process. Since the encoding process of $X_{i,c}$ is the same as that of $X_{i}$, it can be denoted by $z_{i,c}=E_{n}\left(X_{i,c}\right)$, where $z_{i,c}$ is the encoded form of $X_{i,c}$.

The extracted features are used for NLoS identification. Based on problem modeling, we formulated the NLoS identification as a binary classification problem. The GAN is an excellent approach when only LoS data are available [77,78]. On the ond hand, *G* can generate diverse false samples based on noise, facilitating NLoS discrimination in the shortage of NLoS ADCPM samples [79]. On the other hand, with the LoS as the real sample, *D* can accomplish NLoS identification by discerning whether a sample is an LoS sample.

### 3.5. Loss Function

For the DACT-GAN, three losses must be computed: the DACT loss, *G* loss, and *D* loss. The purpose of the AE of the DACT is to fully extract the potential features of the ADCPM samples in the LoS environment, and joint training of the $E_{n}$ and $D_{e}$ is conducted to minimize the reconstruction error. Therefore, we chose the mean square error as a measure of the reconstruction error, which can be expressed as:

(15) $$L_{\mathrm{DACT},\mathrm{AE}}=\frac{1}{B}\sum_{i=1}^{B}{\left(X_{i}-\hat{X_{i}}\right)}^{2},$$

where B denotes the number of samples in the batch.

Considering the presence of $C_{T}$, we want the output of the samples before and after the transformation through AE to be approximate; therefore, we consider the $C_{T}$ part of the DACT loss as:

(16) $$L_{\mathrm{DACT},C_{T}}=\frac{1}{B}\sum_{i=1}^{B}{\left(z_{i}-z_{i,c}\right)}^{2}.$$

By amalgamating the components mentioned above for the DACT loss, we define $L_{DACT}$ as:

(17) $$L_{\mathrm{DACT}}=\nu L_{\mathrm{DACT},C_{T}}+\left(1-\nu \right)L_{\mathrm{DACT},\mathrm{AE}},$$

where ν∈[0,1) denotes the percentage of $C_{T}$-supervised AE feature extraction.

The AE part of the training process is described in Algorithm 1.
**Algorithm 1.** DACT Training Procedure  1:**procedure** Training(batch size *B*)         ▹ Batch number *j*  2:    Clear the parameter gradients of AE and $C_{T}$.  3:    **for** i=1,2,⋯,B **do**  4:        Encode the training sample $X_{i}$ to $z_{i}$.  5:        Decode $z_{i}$ to $\hat{X_{i}}$.  6:        Spatially transform $X_{i}$ to $X_{i,c}$.  7:        Encode the transformed $X_{i,c}$ to $z_{i,c}$.  8:   **end for**  9:   Calculate the loss $L_{\mathrm{DACT}}$.10:   Perform backward-pass.11:   Update the parameters of AE and $C_{T}$.12:**end procedure**

The role of *G* in the GAN is to map a known distribution, which we specify as an unknown distribution, followed by real samples. Conversely, the role of *D* is to discriminate whether the input is a sample generated by a *G* or a real sample. Specifically, *G* generates samples that are as close as possible to the real sample, whereas *D* discriminates as much as possible between the samples generated by the generator, which we call false samples. Thus, they form an adversarial relationship. According to the formulation of the problem, the process of the *D* discriminating between the samples can be categorized as a binary classification problem; therefore, the process can be measured using binary cross-entropy, which can be expressed as follows:

(18) $$\begin{array}{c} \arg\min_{D}\max_{G}E_{X∼p_{data}\left(X\right)}\left\{\log D[E_{n}\left(X\right)]\right\}+E_{n_{g}∼N\left(0,1\right)}\left[\log\left(1-D\left\{E_{n}\left[G\left(n_{g}\right)\right]\right\}\right)\right] \end{array}$$

where we define $n_{g}$ as the original noise conforming to a Gaussian distribution. However, in the actual training process, this adversarial relationship involves alternating instead of simultaneous updates; therefore, the formula can be further decomposed into *D* and *G* losses, where the *D* loss function can be expressed as:

(19) $$L_{D}=\frac{1}{B}\sum_{i=1}^{B}\left[\log\left[D\left(z_{i}\right)\right]+\log\left(1-D\left\{E_{n}\left[G\left(n_{g,i}\right)\right]\right\}\right)\right]$$

and the loss function of *G* can be defined as:

(20) $$L_{G}=\frac{1}{B}\sum_{i=1}^{B}\log\left((1-D\left\{E_{n}[\left(G\left(n_{g,i}\right)\right]\right\}\right).$$

In summary, the training of a GAN can be described using Algorithms 2 and 3.
**Algorithm 2.** Discriminator Training Procedure  1:**procedure** Training(batch size *B*)          ▹ Batch number *j*  2:    Clear the parameter gradients of *G* and the *D*  3:    **for** i=1,2,…,B **do**  4:        Encode the real training sample $X_{i}$ to $z_{i}$.  5:        Input $z_{i}$ to *D* for NLoS identification.  6:    **end for**  7:    Compute $L_{D,\mathrm{real}}$ by comparing the prediction to the label of the real sample.  8:    Perform backward-pass.  9:    **for** i=1,2,…,B **do**10:        Generate Gaussian noise $n_{g,i}$.11:        Generate fake samples: $X_{g,i}=G\left(n_{g,i}\right)$.12:        Encode the generated sample $X_{g,i}$ to $z_{g,i}$.13:        Input $z_{g,i}$ to *D* for NLoS identification.14:    **end for**15:    Compute $L_{D,\mathrm{fake}}$ by comparing the prediction to the label of the fake sample.16:    Perform backward-pass.17:    Compute total $L_{D}$: $L_{D}=\frac{1}{2}\left(L_{D,\mathrm{real}}+L_{D,\mathrm{fake}}\right)$18:    **if** $L_{D}\geq$ Discriminator’s error threshold **then**19:        Update parameters of *D*20:    **end if**21:**end procedure**

**Algorithm 3.** Generator Training Procedure
  1:**procedure** Training(batch size *B*)          ▹ Batch number *j*  2:    Clear the parameter gradients of *G* and the *D*  3:    **for** i=1,2,…,B **do**  4:        Generate Gaussian noise $n_{g,i}$.  5:        Generate fake samples: $X_{g,i}=G\left(n_{g,i}\right)$.  6:        Encode the generated sample $X_{g,i}$ to $z_{g,i}$.  7:        Input $z_{g,i}$ to *D* for NLoS identification.  8:    **end for**  9:    Compute $L_{G}$ by comparing the prediction to the label of the real sample.10:    Perform backward-pass.11:    **if** $L_{G}\geq$ Generator’s error threshold **then**12:        Update parameters of *G*13:    **end if**14:
**end procedure**



Notably, throughout this study, the adaptive momentum (Adam) optimizer was uniformly employed to facilitate the gradient update for each model.

## 4. Simulation Experiments

### 4.1. Scenario Set

To evaluate the effectiveness of the DACT-GAN approach for NLoS recognition, we meticulously simulated the signal propagation in indoor and indoor factory (dense clutter, low BS; hereafter referred to as InF-DL) scenarios, strictly adhering to the 3GPP TR 38.901 standard [19]. The purpose of this study was to test the efficacy of the proposed method under different training and testing scenarios. The channel simulation encompassed four modules: system initialization, large-scale parameter generation, small-scale parameter generation, and multipath coefficient generation. In particular, the time-variant spatial consistency was considered in the simulation to obtain realistic channel characteristics.

First, we considered the common settings. In both scenarios, 272 RBs were allocated to broadcast the SRS with a subcarrier spacing of 120KHz. Thus, $N_{c}=272\times 12=3264$, $N_{f}=2^{\left\lceil \log_{2}\left(N_{c}\right)\right\rceil }=4096$, $N_{t}=144\kappa \cdot 2^{-\mu }=1152$. Notably, $N_{t}$ is defined in terms of 5G time units $T_{c}$, i.e., $N_{t,T_{c}}=N_{t}$, where $T_{c}=\frac{1}{\Delta f_{max}\cdot N_{f,m}}$ with $\Delta f_{max}=480\;\mathrm{KHz}$ and $N_{f,m}=4096$.

Therefore, in systems with different configurations, the number of samples occupied by the CP needs to be multiplied by a coefficient $\zeta =\frac{T_{c}}{T_{s}}$. Based on the above configuration, the coefficient $\zeta =\frac{T_{c}}{T_{s}}=\frac{f_{s}}{f_{c}}=\frac{4096*120*10^{3}}{4096*480*10^{3}}=\frac{1}{4}$ in terms of the system samples; therefore, the length of the CP in terms of the system sample time is $N_{t,T_{s}}=N_{t,T_{c}}\cdot \zeta =1152\times \frac{1}{4}=288$. The transmission power of the UE signal, according to 3GPP TS 38.101-1, is set to 23 dBm, which is the maximum output power for FR1 power class 3 [80]. The SRS itself is configured considering $K_{\mathrm{TC}}=2,\;N_{\mathrm{symb}}^{\mathrm{SRS}}=1$, $C_{\mathrm{SRS}}=61,\;B_{\mathrm{SRS}}=0$, $b_{\mathrm{hop}}=0$, $n_{\mathrm{RRC}}=0$. In addition, we considered that the BS receives the SRS uplink signal to compute the ADCPM, where we set the dimensions of the UPA panel to $J_{g}=I_{g}=8$.

**Figure 4:**
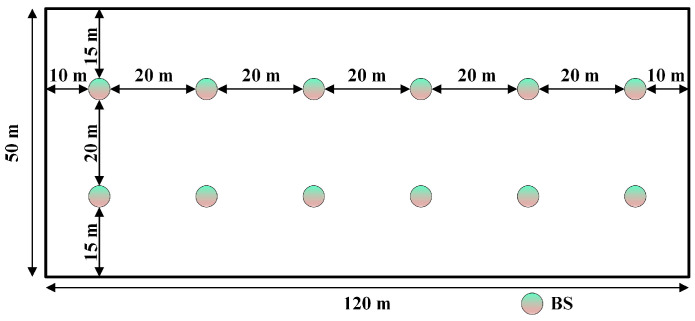
Indoor scenario depicting the distribution of 12 BSs.

Next, we considered the unique characteristics of the different scenarios. For the indoor scenario, an office measuring 120m×50m was set at a ceiling height of 3m. The BS was at the same height as the ceiling, that is, $h_{\mathrm{BS}}=3\;m$. Twelve BSs, spaced 20m apart, were considered. A schematic of the BS positions for the entire scenario is shown in Figure 4. Additionally, the positions of the UE in the scenario were randomly distributed, with the height set to $h_{\mathrm{UE}}=1\;m$. The frequency of the signal transmission carrier was set to $f_{c}=2.565\;\mathrm{GHz}$.

**Figure 5:**
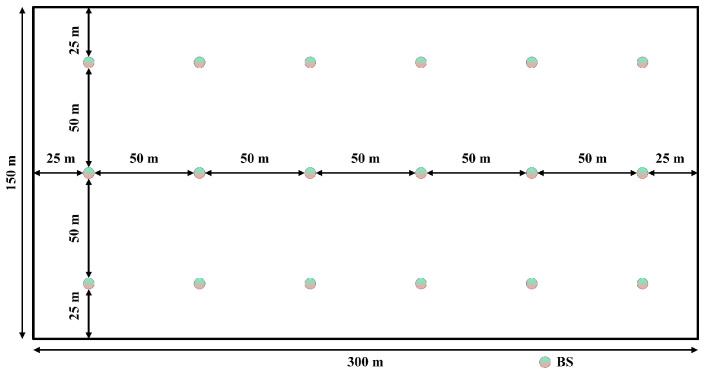
Indoor factory (dense clutter, low BS) scenario depicting the distribution of 18 BSs.

For the InF-DL scenario, the hall size was set to 300m×150m, with a ceiling height of 10m. The BS height was $h_{\mathrm{BS}}=1.5\;m$, with an interstation spacing of 50m; in total, 18 BSs were distributed in the scenario. The height of the UE was $h_{\mathrm{UE}}=1.5\;m$. The signal transmission carrier frequency was set to $f_{c}=3.5\;\mathrm{GHz}$. A schematic of the BS positions for the entire scenario is shown in Figure 5.

For the above scenarios, simulations were conducted for both the LoS and NLoS conditions, ultimately forming four types of datasets. Initially, the SRS signals were generated at the UE end. After channel propagation to the BS end, they were downconverted at the BS. Subsequently, operations such as correlation were conducted to extract the receiver’s H. Subsequently, the ADCPM received at each BS from the UE was obtained through transformation.

### 4.2. Dataset Generation and Testing Platform

Simulations were conducted separately for the two aforementioned scenarios, as well as for the four cases involving LoS and NLoS propagation within these scenarios. Specifically, 65,040 indoor LoS samples were simulated for model training. Additionally, we simulated 5040 indoor LoS samples and 5040 indoor NLoS samples to validate the effectiveness of the proposed method. For the test scenarios, we simulated 35,040 samples each for LoS and NLoS propagation in the InF-DL scenario to evaluate the cross-scenario performance of the method.

The implementation of the test model, along with its training and testing, was conducted using PyTorch 1.10.0 with Python 3.8.18. The simulation platform chosen was equipped with AMD Ryzen™ 5 5600 @ 3.5 GHz, 64 GB of RAM, and NVIDIA GeForce RTX 4060 Ti 16 GB GPU.

### 4.3. Network Components and Specific Parameters

According to the design of the DACT-GAN, the entire network architecture consists of five parts: $E_{n}$, $D_{e}$, $C_{T}$, *G*, and *D*.

The $E_{n}$ and $D_{e}$ were inspired by the architecture design of SegNet [76], with five pairs of symmetric encoding/decoding layers. The encoding/decoding layers extract or restore the features through convolution and perform batch normalization and rectified linear unit (ReLU)-based activation after the convolution. Each encode/decode layer contains two pairs of convolution–BatchNorm–ReLU operations. The first pair is responsible for expanding the feature dimension, while the second pair focuses on hierarchical feature learning. In this paper, the numbers of convolution filters in the $E_{n}$’s convolutional layers are 8, 8, 16, 16, 32, 32, 64, 64, 128, and 128. In contrast, the numbers of filters in the $D_{e}$’s convolutional layers are 128, 64, 64, 32, 32, 16, 16, 8, 8, and 1. All the convolution filters have dimensions of 3×3, with a stride of 1 and padding of 1. Adjacent encoding/decoding layers utilize max-pooling and max-unpooling layers for downsampling and upsampling of the feature maps, respectively. The max-pooling layer employs a 2×2 non-overlapping window with a stride of 2. To implement the maximum unpooling layer, it is feasible to save the pooling indices during the maximum pooling stage and input them into the $D_{e}$. This approach allows upsampling during the deconvolution process based on the indices.

The function of the $C_{T}$ is to perform spatial transformations on the ADCPM. Considering the ADCPM as a one-channel image, we aimed to minimize the effect of the peak positions on the AE feature extraction. Random transformations, such as translation and rotation, must be applied to the images. These transformations fundamentally involved updating the pixel positions and can be mathematically expressed as:

(21) $$\left[\begin{array}{c} x_{i}^{s}\\ y_{i}^{s} \end{array}\right]=A_{\theta }\left[\begin{array}{c} x_{i}^{t}\\ y_{i}^{t}\\ 1 \end{array}\right]=\left[\begin{array}{ccc} \theta_{11} & \theta_{12} & \theta_{13}\\ \theta_{21} & \theta_{22} & \theta_{23} \end{array}\right]\left[\begin{array}{c} x_{i}^{t}\\ y_{i}^{t}\\ 1 \end{array}\right],$$

where $\left(x_{i}^{t},y_{i}^{t}\right)$ represents the pixel index of the transformed image, $\left(x_{i}^{s},y_{i}^{s}\right)$ denotes the pixel index of the original image, and $A_{\theta }$ is the affine transformation matrix. In this study, we adopted the concept of the STN [81], utilizing convolutional and linear layers to extract the image features to obtain the sample-specific $A_{\theta }$. Then, we established a mapping between the source and target images and sampled the original image to generate the transformed image based on $A_{\theta }$.

For the *G* of the GAN, we did not generate the feature map encoded by the AE to facilitate the direct observation of the output of the *G*. Instead, we produced the original ADCPM and passed it through the $E_{n}$. The encoded samples were subsequently fed to *D* for training. We defined the input noise dimension to be the same as that of the encoded ADCPM. The generation process involved four upsampling layers, each comprising a transpose convolution layer, batch normalization layer, and ReLU activation, followed by a final transpose convolution and tanh activation layer to constrain the output range, resulting in the generated ADCPM. In the transpose convolution layers, we set the window size to 4×4, stride to 2, and padding to 1.

For the *D* of the GAN, we initially assumed that the latent features extracted from the ADCPM by the AE would not retain spatial hierarchies. Furthermore, we posited that the AE purified the core features of the ADCPM during feature extraction. Thus, we designed *D* based on an MLP. However, the study did not yield satisfactory results. Consequently, we constructed *D* based on CNNs. Specifically, we built two feature extraction layers: a flattened layer to unfold the feature map and a fully connected layer with a sigmoid function to output the probabilities. The output, due to its sigmoid nature, has a bounded range between 0 and 1. It is reasonable to infer that if the value of $D\left(X_{i}\right)$ is greater than 0.5, the input sample is a LoS sample; otherwise, it is an NLoS sample. In the feature extraction layers, convolution was applied to the inputs with kernel sizes of 2×2 and 1×2, stride of 1, and padding of 0. Subsequently, activation was performed using Leaky ReLU with a negative slope of 0.2. To prevent an overly strong *D* from impairing the training of *G*, we inserted dropout layers between successive convolutional layers to randomly discard the pixels and set the dropout rate to 30%.

Finally, for the AE, the learning rate of the Adam optimizer was set to $lr_{\mathrm{AE}}=0.0001$, with momentum parameters $\beta_{1,\mathrm{AE}}=0.9$ and $\beta_{2,\mathrm{AE}}=0.999$. The learning rates for *G* and *D* were both set to $lr_{G}=lr_{D}=0.0002$, with momentum parameters $\beta_{1,G}=\beta_{1,D}=0.5$ and $\beta_{2,G}=\beta_{2,D}=0.999$.

### 4.4. Baselines

To evaluate the proposed model effectively, we compared it with a series of SOTA models that address the problem of NLoS identification.

- KDE [33]. This method is effective when the sample probability distribution is unknown. The principle of KDE is to fit the LoS distribution by taking a portion of the training sample ${\left\{z_{j}\right\}}_{j=1}^{N_{b}}$ as the baseline for the LoS distribution and then calculating the probability density of the input samples in the LoS distribution, i.e., $\kappa \left(z_{i}|{\left\{z_{j}\right\}}_{j=1}^{N_{b}}\right)=\frac{1}{N_{b}}\sum_{j=1}^{N_{b}}k_{h}\left(z_{i}-z_{j}\right)$. $k_{h}$ is the kernel function with bandwidth *h*; universally, the Gaussian model is used, i.e., $k_{h}\left(x\right)=e^{-\frac{{\left|x\right|}^{2}}{2h^{2}}}$.
- Random Forest [49]. The random forest method, a supervised learning algorithm, necessitates the labeling of NLoS for classification learning. It uses bagging to partition the training set into several subsets and train numerous decision tree models. Test samples are dispatched to several decision trees for analysis, and the model consolidates the classification outcomes from all decision trees and votes to ascertain whether it is NLoS propagation.
- AE-KDE [68]. This approach consists of two steps. Initially, train an autoencoder to thoroughly investigate the latent properties of the training samples. Subsequently, a selection of the encoded samples is utilized as the baseline, and the KDE approach is employed to ascertain if the test sample conforms to the LoS probability distribution, hence indicating whether it represents LoS propagation.
- GANomaly [71]. This model utilizes a generator network composed of an encoder–decoder–encoder architecture with a discriminator that evaluates the latent features encoded from both samples and their reconstructions. Because the training process involved only LoS samples, the input of the NLoS samples caused significant reconstruction errors, thereby enabling the effective identification of NLoS during the testing phase.

## 5. Results and Discussions

### 5.1. Data Processing

**Figure 6:**
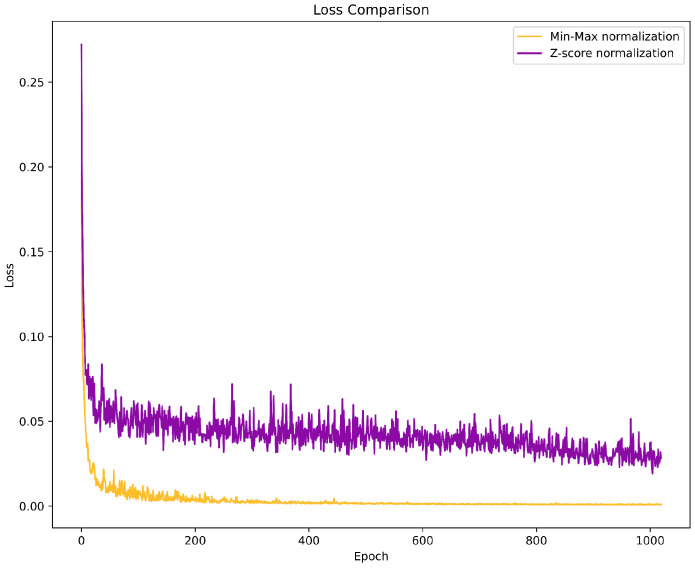
Comparative effects of different data normalization techniques on model training efficacy.

Data preprocessing plays a crucial role in scenario testing, where various factors, including the distance between the terminal and signal source and the presence of obstructions, can influence the magnitude of the channel samples, thus making it necessary to preprocess the training and testing samples for more accurate discrimination. The two standard methods are min-max normalization and Z-score normalization. Min-max normalization scales all sample values by obtaining the maximum value in the data, ensuring that all values fall within the range of [0, 1]. In contrast, Z-score normalization first calculates the variance and expectation of the sample values and then applies the transformation $\left(\frac{x-\mu }{\sigma }\right)$ to standardize the samples to a mean of 0 and variance of 1. Through testing, we found that min-max normalization significantly outperformed Z-score normalization. One explanation for this result is that the $E_{n}$ in this study was designed based on a CNN, which utilizes ReLU as the activation function in the hidden layers, whereas using Z-score normalization may result in the loss of some data information. Figure 6 illustrates the variation in the model training efficiency for different data normalization methods.

### 5.2. Batch Size

**Figure 7:**
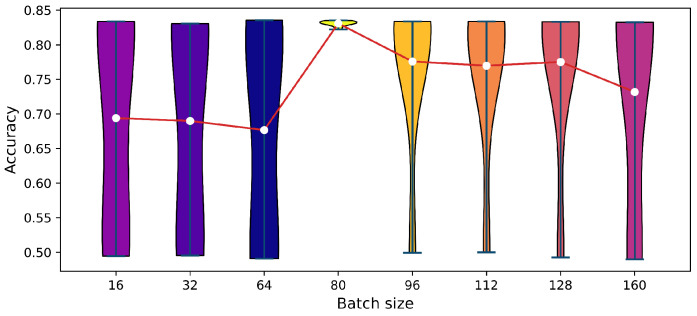
Violin plots comparing the training accuracies of DACT-GAN across different batch size *B* with the mean training accuracy for each batch size *B*.

In the process of training the DACT-GAN, adjusting the batch size has a significant influence on the stability and effectiveness of model training. In Figure 7, we analyze the NLoS identification accuracy that DACT-GAN can achieve after training for 20 epochs when the batch size $B\in \left\{16,32,64,80,96,112,128,160\right\}$. We observed that the training was unstable when *B* was relatively small. We attribute this to the increased variance in the gradient estimation and instability in the model update direction during the AE training process with a smaller *B*. Furthermore, a smaller *B* tended to cause oscillations in the adversarial game between *G* and *D*. However, when *B* was larger, although the instability of the model was noticeably improved, an excessively large *B* weakened the generalization ability of the AE for new data, which is undesirable for cross-scene NLoS recognition. Consequently, considering the stability and generalization capability of the model as well as the consumption of computational resources and training duration, we ultimately determined B=80 as the optimal batch size.

GAN architectures differ from conventional network designs, necessitating adversarial training between *G* and *D*. Successful network training cannot be achieved if either entity is too strong or weak. Instead of simply adjusting the learning rates, we employed two strategies to ensure stable GAN training and achieve the highest possible accuracy.

### 5.3. Label Smoothing

**Figure 8:**
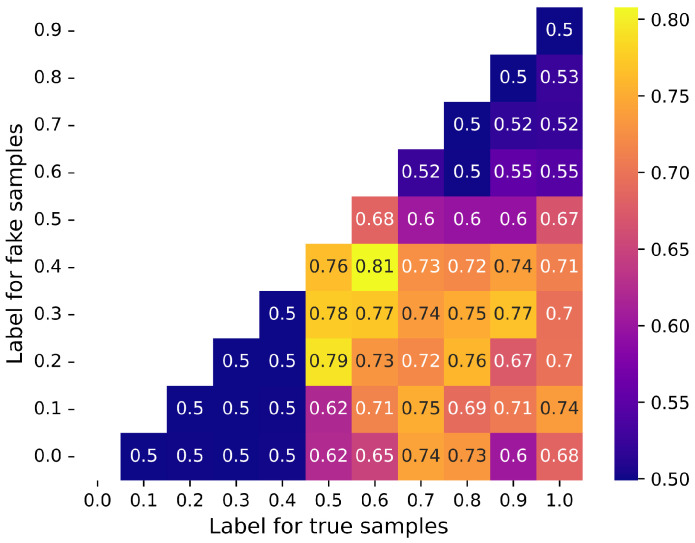
Heatmap of accuracy with label smoothing applied at different thresholds for G and D.

First, we adopted the label-smoothing technique to prevent *D* from confidently predicting the labels, which could cause *G* to produce highly similar or identical samples, a phenomenon known as mode collapse. By decreasing the values of the real sample labels and increasing those of fake labels, we moderate the learning process of *D*, enhancing the model’s generalization capability for unseen samples, and encourage *G* to produce more diverse samples. To determine the optimal real and fake sample label values, we iterated over all possible pairs of real and fake sample labels within the range $\left[0,1\right]$ using 0.1 as the step size, across ten trials with training cycles of 20 epochs each. Scenarios in which the real sample label values are less than those of the fake samples are illogical and were, therefore, disregarded. As shown in Figure 8, the results indicate that the training is unstable when the real sample label is 1 and the fake sample label is 0, because of the *D*’s overconfidence.

Conversely, when both labels are low, the GAN training collapses and cannot be completed. Similarly, when both labels are high, *G* fails to complete the training because it hastily deems the samples as real. Ultimately, we deduced from the graph that the system achieves optimal performance when the real sample label is set to 0.6 and the fake sample label is set to 0.4.

### 5.4. Early Stopping

The second strategy for training a GAN involved early stopping. Considering the training of GANs required an adversarial process between *G* and *D*, it could destabilize the training process if either operation becomes too dominant. Therefore, we set loss thresholds for *G* and *D*, halting training when the loss of either *G* or *D* fell below these thresholds. This method aimed to maintain a dynamic balance between the training processes of *G* and *D* and saved computational resources and training time to a certain extent.

**Figure 9:**
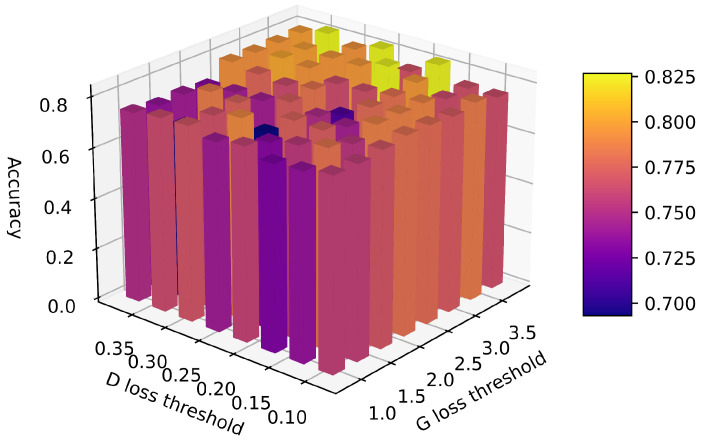
Three-dimensional bar chart showing the effect of early stopping on DACT-GAN accuracy across various loss thresholds for *G* and *D*.

In this study, we set the generator’s loss threshold in the range $\left[0.8,4\right]$ with a step of 0.4, and the discriminator’s loss threshold in the range $\left[0.08,0.4\right]$ with a step of 0.04. These threshold ranges were selected based on the loss values of both the *D* and *G* when the early stopping strategy was not employed. The results, as illustrated in Figure 9, indicate that appropriately increasing the loss threshold of *D*, as opposed to that of *G*, can significantly enhance the training precision. This inference is based on the premise that the *D* is prone to overfitting in this context. Overfitting by *D* prevents the *G* from determining suitable gradients to improve the model. From the graph, we conclude that the system achieves optimal performance when the generator’s loss threshold is set to 3.2 and the discriminator’s loss threshold is set to 0.2.

### 5.5. Baseline Parameter Selection

**Figure 10:**
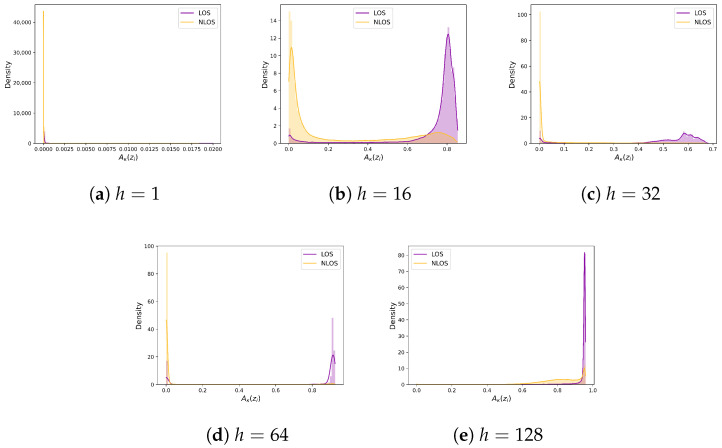
Classification of NLoS and LoS after 20 epochs of training with different values of h of AE-KDE.

This experiment aims to illustrate the effect of the AE-KDE parameter selection on the results of the baseline, which involves KDE, unlike other hyperparameters that only affect the training phase. The number of samples considered and the choice of kernel bandwidth *h* during KDE can affect the estimation performance. For this purpose, we conducted training and testing by setting different bandwidth values *h*, assuming the original sample size for KDE is 2048, with $h\in \left\{1,16,32,64,128\right\}$. The resulting LoS and NLoS distribution diagrams are shown in Figure 10. The horizontal axis represents the KDE output of each data sample. When the value of h is minimal, the distributions of LoS and NLoS cannot be separated, with both trending toward zero.

Conversely, when *h* is too large, NLoS identification becomes ineffective as the overall outputs of the LoS and NLoS samples tend toward 1. Ultimately, the separation effect between the LoS and NLoS samples was found to be the best when h=64, which was also used in subsequent comparative experiments. Furthermore, comparing the range of values indicated that the optimal decision threshold changed with variations in the *h* values. Therefore, although the AE-KDE method can generate an optimal distribution, its threshold selection is not fixed and poses certain recognition risks.

### 5.6. Computations and Memory Access Requirements

Recognizing NLoS situations is essential for terminal positioning computations in industrial location-based applications, especially in high-risk processes, where real-time updates of positioning data are vital for worker safety. Consequently, the model’s prediction speed is paramount, with swifter values being unequivocally superior. Consequently, we computed the necessary calculations and memory accesses for both the comparative technique and the proposed way. We utilize one ADCPM as the input sample.

To illustrate this table, we have derived the computational complexity and the amount of memory required for the KDE method. Initially, the kernel function necessitates the computation of the Euclidean distance between the test sample and the samples from the specified distribution, resulting in two floating-point operations per dimension: one for the difference and one for the square. Due to the differing sample dimensions of KDE and AE-KDE, the sample dimension is provisionally established as ι for presentation purposes. A total of 2ι operations are necessary to compute the difference and the square of each dimension. Following the computation of the square of the difference in each dimension, it is essential to aggregate all dimensions, necessitating ι−1 operations. Ultimately, dividing by the coefficient, executing an exponential function operation, and subsequently normalizing necessitates around 17 operations. In conclusion, each kernel function necessitates 3ι+16 operations. Ultimately, by multiplying the number of test samples by the number of samples from the specified distribution, one can ascertain the computational requirements for the KDE approach. In this study, we specify that the sample size in the given distribution is 2048, necessitating 2048×(3ι+16) operations. The memory utilized is predominantly expended while accessing the test samples and those inside the specified distribution during the computation of the kernel function, indicating that the memory usage is 8192ι bytes.

Table 1 indicates that DACT-GAN has the minimal memory access requirement, as it does not necessitate temporary sample storage, relying solely on *D* for NLoS recognition throughout the testing phase. *D*’s simplified architecture results in the lowest processing requirements among machine learning approaches. The computational complexity of AE-KDE is analogous to that of DACT-GAN. Nevertheless, the application of the KDE technique requires the provisional retention of certain samples from the specified distribution, leading to increased memory consumption. Nonetheless, due to the encoding of the temporarily stored samples, the memory access demand is not excessive.

GANomaly requires greater computational resources because of its encoder–decoder–encoder architecture. Consequently, GANomaly’s computing demands significantly exceed those of DACT-GAN and AE-KDE. Ultimately, the KDE technique does not engage in neural network activities, resulting in minimal computational demands. Nonetheless, due to its continual necessity to access a substantial quantity of original samples, KDE’s memory access demands are the most significant among all methodologies.

It is important to recognize that random forests, being constructed from decision trees, primarily utilize comparative situations to ascertain LoS and NLoS. Consequently, the calculated amount is not comparable to that of other methods and is not presented in this table.

### 5.7. Performance Comparison

To objectively assess the performance of our proposed DACT-GAN, we trained our method and the baseline using LoS data from an indoor scenario. We performed tests using equal LoS and NLoS samples in the InF-DL scenario. Table 2 lists the average area under the curve (AUC), F-score, Accuracy, Precision, and Recall after training for 20 epochs.

We discovered that the KDE method signifies the lower bound. This outcome arises from the substantial variation in the ADCPM peak’s position relative to the distance and angle between the BS and UE in the test sample. Nevertheless, the KDE technique directly computes the Euclidean distance between the two ADCPMs. Consequently, it is easy to misinterpret LoS samples. Due to its utilization of many decision trees, the Random Forest technique can more effectively ascertain whether a sample qualifies as a LoS sample compared to KDE. Consequently, it attains superior performance compared to KDE. However, this method requires labeled NLoS training samples in practical applications, so it needs further consideration from the practical use perspective.

In the realm of unsupervised learning techniques, GANomaly considerably surpasses conventional machine learning and statistical approaches. The encoder effectively extracts the latent features of ADCPM. Nonetheless, GANomaly is heavily reliant on the data and presents considerable difficulties in enhancing performance due to the information loss associated with the encoding–decoding–encoding process.

In contrast, AE-KDE achieved the second-best performance in this test because the AE effectively learned the latent features of the LoS samples, and the KDE method learned and analyzed the probability density distribution of the LoS samples. Moreover, a log function was used to amplify the differences in probability density. However, this estimation is based on Gaussian kernel fitting, and the actual LoS propagation cannot be strictly assumed to follow a Gaussian distribution. Hence, perfect estimation cannot be achieved.

Finally, DACT-GAN outperformed the other two methods, partly because during the training of the AE, we diminished the influence of the ADCPM peak positions on feature extraction in the loss function, allowing the model to focus more on the latent features of the LoS samples. However, in comparison with the KDE method, the GAN is more adaptable to complex distributions that are difficult to describe using the existing mathematical models, thus achieving the best results in this test.

## 6. Conclusions

This study investigated the NLoS propagation identification problem in implementing high-accuracy positioning for 5G. First, we defined a 5G system model based on massive MIMO, analyzed the propagation of the uplink SRS under LoS conditions, and adopted the ADCPM transformation method as a sparse input to the network. Given the variability and unpredictability of NLoS, we used the LoS samples as the sole input to the network and innovatively proposed the DACT-GAN method. This method utilizes an AE to extract the features from the ADCPM while employing a $C_{T}$ to circumvent attention shifts caused by spatial location changes. Subsequently, we constructed a GAN in which fake samples generated by *G* introduce diversity into the training process, thereby enhancing the training of *D*, which ultimately serves as the NLoS identifier. Moreover, during the online phase, only the $E_{n}$ and *D* were used, significantly reducing the computational demands on the devices.

We conducted signal propagation simulations for the proposed method under 3GPP standards in both indoor and InF-DL scenarios. By separating the training and testing scenarios, we aimed to confirm the cross-scenario robustness of our proposed method. The test data showed that the proposed DACT-GAN is more effective than the SOTA DL models, requiring only 87.3% and 33.6% of the inference time of AE-KDE and GANomaly, respectively. Moreover, unlike the KDE method, our approach does not require fixed storage of many original distribution samples in the GPU memory during the online phase, significantly reducing the device resource demand. Furthermore, the DACT-GAN method addresses the issue of SOTA methods, wherein the identification threshold varies with the training hyperparameters or scenario changes. Finally, our method improved the performance by 2.15% and 8% compared to AE-KDE and GANomaly, respectively.

Mobile communication networks, poised to evolve into vast distributed neural networks in the upcoming 6G era, will integrate communication, perception, and computing capabilities, extending from human connectivity and the Internet of Things to comprehensive intelligent connectivity for everything. At the same time, semantic coding based on multimodal information such as images, voice, and text will gradually replace traditional source coding. Therefore, efficient and accurate image information extraction is an important research direction.

When it comes to cellular positioning, neural networks’ hardware foundation will enable communication networks to have ML capabilities. The development of the Internet of Everything has created a demand for positioning from numerous nodes within the industrial Internet to home local area networks. High-precision positioning is even more necessary for applications such as safe production and elderly care. In complex environments such as factories and indoors, non-line-of-sight signal propagation can seriously affect the measurement of arrival time, which in turn affects positioning accuracy. As a result, the NLoS recognition method proposed in this paper, based on deep learning, distinguishes between LoS and NLoS signal propagation from a recognition perspective. Moreover, the lower complexity allows nodes with lower power consumption in the industrial Internet to also complete NLoS recognition.

In subsequent research, we will explore NLoS suppression methods to deal with situations where the positioning source is insufficient for positioning after NLoS recognition. On the other hand, we will also explore multimodal positioning, combining information such as images and poses to compensate for the shortcomings of wireless positioning and achieve more reliable and higher-precision positioning to empower safety applications.

## Figures and Tables

**Table 1:** Computation and memory access requirements for baselines and DACT-GAN.

	Computational Requirements (M FLOPs)	Memory Access Requirements (M Bytes)
DACT-GAN	156.4420	1.3303
AE-KDE	179.0490	20.0560
GANomaly	465.8480	3.5348
KDE	113.2790	155.9950

**Table 2:** Comparison of mean performance indicators across 10 trials: proposed DACT-GAN vs. Baselines.

	AUC	F-Score	Accuracy	Precision	Recall
DACT-GAN	0.8299	0.8289	0.8299	0.8371	0.8299
AE-KDE	0.8124	0.8103	0.8124	0.8271	0.8124
GANomaly	0.7684	0.7684	0.7684	0.7686	0.7684
Random Forest	0.7152	0.6926	0.7152	0.8047	0.7152
KDE	0.6916	0.6592	0.6916	0.8090	0.6916

## Data Availability

The original contributions presented in the study are included in the article, further inquiries can be directed to the corresponding authors.

## Appendix A. Proof of the Far-Field Assumption in This Paper

This study considered the propagation of 5G signals in a sub-6 GHz environment. The far-field assumption is valid when the distance *d* between the antennas is significantly greater than the wavelength of the electromagnetic wave, with the empirical threshold being twice the wavelength. Based on the simulated scenarios, two propagation environments were considered: InF-DL and indoor. Considering the fixed height of the UE but with random positioning, only the height difference was evaluated. In the InF-DL scenario, the BS height was $h_{\mathrm{BS}}=1.5\;m$, and the UE height was $h_{\mathrm{UE}}=1\;m$, resulting in a height difference of d=0.5m. In this scenario, with the center frequency of the 5G signal set at $f_{c}=3.5\;\mathrm{GHz}$, the wavelength $\lambda =\frac{c}{f_{c}}=0.086\;m$ according to the wave equation λf=c, where *c* is the speed of light. Thus, d/λ=5.81>2, satisfying the far-field assumption.

Similarly, for the indoor scenario, with the BS height at $h_{\mathrm{BS}}=3\;m$ and UE height at $h_{\mathrm{UE}}=1\;m$, the height difference was d=2m. With a signal center frequency of $f_{c}=2.565\;\mathrm{GHz}$, the wavelength is λ=0.12m, and d/λ=16.67>2, also meeting the far-field assumption.
